# Stakeholder perspectives on a hypothetical rapid test for antibiotic resistant bacteria: an exploratory study

**DOI:** 10.3389/frabi.2025.1729093

**Published:** 2026-01-12

**Authors:** Kelly Laas, Kimberly Vargas Barreto, Elisabeth Hildt

**Affiliations:** 1 Center for the Study of Ethics in the Professions, Illinois Institute of Technology, Chicago, IL, United States; 2 Providence St Joseph Health, Renton, WA, United States

**Keywords:** self-testing, antibiotic resistance, ethical implications, benefits, risks

## Abstract

Antibiotic resistance remains a significant public health concern. One possible solution is to develop a new type of highly accessible test for antibiotic resistance that can be rapidly and easily utilized. As new diagnostics for measuring antibiotic resistance continue to be developed, several key practical, ethical, and social factors must be considered, including the types of tests that might be useful, their potential beneficiaries, and the contexts in which they should be utilized. This study aims to gather insights from key stakeholders regarding the ethical implications, benefits, and potential risks associated with a hypothetical rapid antibiotic resistance test that may also be designed for home use. A total of 32 semi-structured interviews were conducted with three stakeholder groups: potential users, medical providers, and ethicists. While prospective users of the test were generally positive about the proposed test, this might reflect public acceptance of point of care/home tests in general, rather than one specifically measuring ABR. Medical providers and experts knowledgeable about the problems of antibiotic overuse quickly pointed out some drawbacks and areas of concern for home testing for ABR, offering helpful guidance on where further research and consideration are needed.

## 1 Introduction

The continued rise in resistance to antibiotics in human bacterial pathogens is a significant problem for human health (Jamrozik and Heriot, 2022; Ferri et al., 2017). In 2019, it is estimated that antibiotic-resistant bacteria (ABR) were responsible for about 1.27 million deaths and contributed to an additional 4.95 million deaths worldwide (Murray et al., 2022). Low and middle-income countries suffer the highest instances of infections and deaths from antibiotic-resistant bacteria, likely due to the lack of surveillance for ABR strains. The harmful effects of this include the need to use newer, more expensive medications to replace now-ineffective first-line antibiotics, a longer duration of illness, and treatment-related costs associated with antibacterial resistance, which increases healthcare costs and the economic burden on patients and societies (World Health Organization, 2025). The harmful effects of antibacterial resistance (ABR) are unequally distributed, falling disproportionately on those with limited access to clinical testing.

The improper use of antibiotics is a leading cause of antibiotic resistance in humans. This includes prescribing antibiotics unnecessarily for viral infections and using broad-spectrum antibiotics that kill non-target bacteria along with the intended pathogen. The level of antibacterial resistance is higher with broader-spectrum antibiotics and seems to increase with longer durations of antibiotic treatment (Morley et al., 2019). In countries where antibiotics are available without a prescription or used as additives in livestock farming to promote growth, ABR tends to develop rapidly (World Health Organization, 2023).

Bioethicists discussing this problem sometimes frame it as a “dual agent” problem, in which prescribers of antibiotics are responsible for *both* the patient with an infection that needs treatment and the wider community, which requires antibiotic stewardship (Littmann et al., 2018; Jamrozik and Heriot, 2022). Against this background, antibiotic resistance has been discussed as a tragedy of the commons - a concept in which the use of a resource by individuals who benefit from it leads to negative implications for everyone (Hays et al., 2022; Giubilini, 2019; Jamrozik and Selgelid, 2020; Jamrozik and Heriot, 2022). The off-target use of antibiotics often occurs when clinicians prioritize the immediate needs of their patients over the interests of other, distant, or future patients. This is typically seen in healthcare settings, where broader-spectrum empiric antibiotics are used instead of narrower ones (Khurana et al., 2024), as is sometimes required by prevailing medical guidelines, such as in the treatment of severe sepsis in hospital settings (Sepsis Alliance, 2025; Rhee et al., 2024).

Monitoring of ABR at the national, local, and even hospital or clinic levels is crucial to ensure the optimal therapeutic option for treating bacterial infections (van Belkum et al., 2019). Tests are available at both the group/population level to measure resistance trends and at the individual level to select the best antibiotic for a particular patient. Standard individual tests are currently used in clinics and hospital settings, including agar dilution, broth microdilution, and disk diffusion, which involve exposing isolated bacteria to antimicrobial agents and assessing their growth (Elbehiry et al., 2025). While these approaches are cost-effective, they are also highly labor-intensive and can delay treatment (Maugeri et al., 2019; Shin et al., 2019). New tests would be useful hat are both rapid, accessible, and accurate to quickly alert medical professionals to the presence of ABR and enable targeted treatment.

New technologies must meet the challenges of sensitivity, accuracy, reproducibility, rapid turnaround time, simplicity, and affordability (Dietvorst et al., 2020; Mo, 2022). The search for functional rapid tests for ABR is currently underway, focusing on developing sensors and systems that accurately guide physicians to the most effective antibiotic for prescribing in an infection (Leonard et al., 2018). As the availability of medical diagnostics increases, care becomes more widely dispersed, with some tests, such as for COVID-19, becoming available at pharmacies and even at home (Howard-Wilson et al., 2024), One question arises in this context. Is there a need for an easily accessible rapid test for antibiotic-resistant bacteria? Perhaps even one that patients can administer? And if one were to be developed, what are some of the ethical questions that loom large in its research, development, and potential use?

Understanding public perceptions can increase awareness and knowledge of the status quo and help develop strategies for improvement. One possible solution to existing issues with ABR is to develop a highly accessible test that can be rapidly and efficiently utilized. Input from relevant stakeholders can help design such a test, improving early detection and management of ABR bacteria and, in turn, reducing transmission, lowering healthcare costs, and preventing misuse of antibiotics. Thus, ultimately contributing to public health and safety. Moreover, by investigating stakeholders’ views on the conception of such a test, we can ensure their participation in the initial development process, i.e., idea generation, as understanding potential users’ preferences and concerns can lead to systems that respect autonomy and support informed decision-making. Lastly, by collecting and analyzing initial attitudes across stakeholder groups, we can help identify knowledge gaps or misinformation about ABR and rapid tests before they are developed. This can lead to targeted education campaigns, fostering a more informed public that is better equipped to make health-related decisions and address concerns for epistemic injustice or knowledge gaps amongst populations.

This study proposed a hypothetical test to different stakeholders to gain insight into its benefits, risks, and significant ethical questions. At this point, a rapid, point of care ABR test is an emerging technology, namely one that is at the very early stage of development, and no products are on the market or are being used by clinicians or ordinary users. Therefore, many questions about its nature, future use, and social consequences are still undecided (Brey, 2011). This interview study falls within the tradition of “moral experiments,” in which data is gathered through experimental methods to investigate moral judgments, reasoning, and behavior (Alfano et al., 2022). Exploratory moral experiments of this kind aim to examine the ethical issues raised by a new technology (Van de Poel, 2017). Specifically, interviewees are asked about a hypothetical technology that does not currently exist to anticipate the ethical issues, risks, benefits, and questions that should be considered in the conception, development, and use of an emerging technology.

In 2023, the Walder Foundation funded a research project looking at the potential of developing a “measurement in place” or “lab in a vial” test that could be used to detect ABR. The concept behind this was to enable users to get information about their health and to track health-related aspects of themselves and the environment. The interview study is part of a larger project conducted by an interdisciplinary team at the Illinois Institute of Technology in collaboration with researchers from Northwestern University and the University of Chicago in the Chicago area. The study had two goals. One goal was to take steps towards developing a rapid test for antibiotic-resistant bacteria. The ethics sub-project, discussed here, focused on the perspectives of various stakeholder groups regarding the benefits and potential risks of rapid tests, such as those for antibiotic-resistant bacteria.

This interview study examines public perceptions about the potential development and use of rapid tests for antibiotic-resistant bacteria, specifically point-of-care tests (i.e., medical diagnostic tests performed at or near the site of patient care), as well as patient-administered or home tests (lab in a vial/measurement in place). This type of test was used as an example of a test that could measure health-related aspects of their environment in a cost-effective and easy-to-conduct manner.

By researching these perspectives, we aim to gain a deeper understanding of the relevant aspects for development and potential adoption of ABR rapid tests. We were interested in stakeholder views on ethical issues connected to antibiotic-resistant bacteria in general. Additionally, we asked questions about where and how such a rapid test should be delivered, i.e., at home or in a clinic.

A different part of the interview study, which takes a broader perspective and examines stakeholder perspectives on environmental health-related tests, has already been published elsewhere (see Laas et al., 2025).

## 2 Materials and methods

### 2.1 Data collection

The research team for this project consisted of three ethicists: a postdoctoral scholar specializing in bioethics, a professor of philosophy and applied ethics, a librarian with 20 years of experience in applied ethics, and a biomedical graduate student serving as a research assistant at the Ethics Center.

In October-December 2023, the research team conducted a semi-structured interview study to understand what potential users, medical providers, and ethicists perceive as the main ethical implications, benefits, and possible risks of rapid antibiotic-resistant bacteria tests. The interview consisted of two parts, the first focusing on potential concerns and benefits of a rapid test for antibiotic-resistant bacteria. The second part, discussed in an earlier paper, centered on the interviewees’ views on environment-related testing.

The interview was organized in the following way. After the demographic questions, the interviewee was informed about the study and told that the project was focused on developing “a method for swiftly detecting antibiotic-resistant bacteria or other similar infections without sending biological samples to a lab.” The interviewer went on to describe how this hypothetical test differs from current testing methods. “Current methods for detecting antibiotic-resistant bacteria can take 24–48 hours under the best circumstances. The goal is to develop a rapid test for detecting antibiotic-resistant bacteria. This should enable potential users to receive treatment promptly, rather than waiting for lab results to be returned. The proposed test could be either at a doctor’s office, health clinic, or potentially even administered at home by the potential user, somewhat like a COVID test.” Due to the ongoing nature of the test being developed by the biomedical research team, we were unable to provide specific details about this hypothetical test, such as a use case, the manner of sample collection, or a prototype concept. This is a major limitation of our study, and results should be viewed in this light.

Two versions of the interview questionnaire were developed: one for healthcare providers and one for potential users and ethicists, to reflect the different viewpoints and insights each group might provide. Interviewees fell into the following categories. 1) Ethicists – individuals who hold at least a master’s in philosophy, and related coursework, or publications in the field of bioethics. 2) Medical professionals – individuals who hold an MD/PhD and see patients in a medical context and/or teach at a medical institution. 3) Potential users – individuals over the age of 18 with varied degrees of education, who have some experience using medical services, either in the U.S. or abroad. Of interviewees, four had a chronic condition, 25% self-reported visiting a medical doctor’s office every 3 months or more, and 75% reported visiting a medical doctor around once a year. The questionnaire, which was developed as a unique measure, was validated by sharing the final draft of the test with experts in the field and colleagues who fit the main stakeholder profiles of potential interviewees. The final draft of the questionnaires was edited according to this feedback, and prompting questions and definitions were developed to help explain terms that might not be readily understandable. (See Appendix I in the **Supplementary Materials** provided).

IRB approval was obtained for the study questionnaire and recruitment material. The team reached out to potential interviewees through flyers on campus and in the surrounding Chicago area, as well as via the university’s campus newsletter, social media, and the Ethics Center’s international mailing list. The research team opted for a snowball sampling method to ensure the sample encompassed a broad spectrum of perspectives.” Throughout the interview, interviewees were provided with definitions of key terms such as antibiotic resistance. The interviewers avoided using complex medical terms unless the interviewee introduced them into the discussion, and interviews were frequently paused to allow for explanations to be sure that terms were fully understood. Interviewees received a $25 Amazon gift card for participating. The interviews were conducted over Zoom video calls and recorded. The transcription produced by the Zoom software was then edited for clarity and to remove identifying information. In total, thirty-two interviews were conducted with 14 potential users, 11 medical providers, and 7 ethicists.

The demographics of the interview participants are presented in **Table 1** below.

**Table 1:** Interviewee demographics.

Characteristics	Number	Percentage
Interviewee Category		
Medical Provider	11	34%
Ethicist	7	22%
Potential User	14	44%
Gender		
Male	15	47%
Female	17	53%
Ethnicity		
Caucasian	14	44%
African/African American	7	22%
Asian/South Asian	8	25%
Hispanic	1	3%
Multiple Ethnicities	2	6%
Age		
18-30	10	31%
31-45	11	34%
46-65	8	25%
65+	3	9%
Education Level Achieved		
High School	2	6%
BA	6	19%
Masters	9 (of these, 5 pursuing a PhD/MD	28%
Ph.D./MD	15	47%
Nationality		
United States of America	13	41%
European (Germany, Estonia, Netherlands, Poland)	9	28%
Africa (Nigeria)	5	16%
South Asia (India, Pakistan)	3	6%
Mexico	1	3%
Asia (South Korea)	1	3%
Do you have a chronic health condition?		
Yes	4	13%
No	28	87%
How many medical visits		
Once a month or more	2	6%
Once every three months	6	19%
Once a year or less	24	75%

### 2.2 Data analysis and methodology

In January 2024, the anonymized and edited final transcripts were loaded into Taguette, an open-source qualitative research tool, to facilitate easy coding and analysis (Rampin and Rampin, 2021). The team used grounded theory methodology to analyze the interviews. Therefore, three team members independently coded three randomly selected transcripts from each participant group, then compared and discussed the findings across the groups. From this, the team developed a shared coding method, with categories emerging through successive levels of analysis and discussion. Following this, two team members coded each interview separately and then discussed the results as a group. Ultimately, 49 codes were generated for analyzing the entire interview.

Section three that presents the results is organized as follows: 3.1 discusses the interviewee responses concerning the benefits of the test, 3.2 addresses interviewees’ concerns about the test, 3.3 examines aspects related to the reliability, accuracy, and specificity of the proposed test, 3.4 explores answers that discuss the affordability, accessibility, and potential influence on health disparities that the test may have, 3.5 looks at responses on where the test should be taken, 3.6 discusses answers on advice or support need to test interpretation, 3.7 presents thoughts on sharing test results, 3.8 talks about interviewees’ views on similar rapid, home tests for sexual transmitted infections, and 3.9 discusses what interviewees thought that medical providers need to know about the test. Finally, 3.10 summarizes answers to questions explicitly asked to medical providers and ethicists, and 3.11 summarizes topics that came up frequently in all interviews, regardless of the question being asked.

## 3 Results

### 3.1 What do you think might be the benefits of using a test like the one proposed?

All interviewees answered this question, but replied in several ways and mentioned different types of benefits, including being more exact about the kinds of antibiotics needed to treat an infection, rather than having to wait for a culture to come back from the lab, decrease healthcare costs, being more convenient and rapid, and potentially a decrease in antibiotic resistance in the overall population.

The most frequently used codes in answering this question included rapidity of test (23 respondents), early detection (14), convenience (10), accessibility (9), and affordability (6).

Examples of interviewees who stressed the rapidity of the test and the benefits of early detection:

“[The] benefits of high speed by just knowing if there is anything wrong with you, knowing what exactly it is. I think that’s a huge benefit soon[er] rather than later. It allows you to be proactive in your health.” (Int. 26 -potential user).

“I think being able to act quicker is especially important … [for] babies and infants. They often suffer from infections that can’t be treated because we don’t know the pathogen. So that would enable them to treat them quicker and hopefully decrease the death rates of babies.” (Int. 23-medical).

Four respondents also focused on risk when answering the question about potential benefits, raising questions about the accuracy, sensitivity, and reliability of the rapid test, correct specimen use, affordability, and the possible impact on lab technicians’ jobs.

### 3.2 Benefits

Eight individuals spoke about the benefits of the test beyond the question discussed above, including six medical professionals, one ethicist, and one potential user. Interviewees shared their thoughts about the benefits of using the test in the clinic, provided users were willing to pay for it, and highlighted the need for support in interpreting the test results. The codes that were mentioned most frequently in conjunction with benefits were accessibility (3) and affordability/decreased healthcare costs (2).

“I think it’s a good thing if they develop it. Especially in developing countries. There’s so many hospitals where people don’t have access to antibiotics. They don’t have labs that are working properly. There’s people who are not insured. So they’re mainly paying out of pocket. It’s gonna be very beneficial for those people if something cheaper [is available].” (Int. 29-medical).

#### 3.2.1 What concerns would you have in using a test like the one proposed here?

Twenty-five respondents answered this question. In a handful of cases, the respondent preemptively discussed possible concerns about benefits, which led the interviewer to skip asking this question a second time. The codes that appeared the most in answer to this question were accuracy (12), sensitivity/specificity (9), reliability (8), potential misuse of the test (5), diagnosis and use (3), specificity (3), affordability (3), interpreting test results (3), follow up care/action needed (3), withdrawal from medical consultation/self-care (2), antibiotic overuse (2), follow up care/action required (2), and privacy (2).

Medical providers and ethicists expressed particular concern about the test’s accuracy, sensitivity, and reliability, and many commented on the potential for misunderstanding by untrained users.

“I think it depends on the field. I would suppose that if you were to just sell it to everyone, also non-medical professionals, I would think it could cause panic, and they would come to conclusions about their health that are not necessarily true. And also, maybe demand treatment where it’s not needed,” (Int. 23-medical).

Potential users were also concerned about the test’s reliability.

“I’m not in the medical line, but as patient, I’d be worried about, can this test actually show the ailment I’m suffering from? I’m not going to the traditional laboratory the way we are used to and I’m now introduced to this new technique. So I should be concerned? Can this actually show the bacteria I have?” (Int 5-potential user)

#### 3.2.2 Concerns/risks

Thirteen individuals commented on concerns/risks beyond the question, specifically regarding concerns they had about the test, including 6 medical professionals, 3 experts, and 4 potential users. Interviewees primarily discussed concerns when answering questions about home testing (6), the reliability of the test (6), and obtaining support when interpreting the test results (3).

Codes that came up most frequently in conjunction with “concerns/risks” included test interpretation (10), comparison to current lab practices (7), possible consequences of using the test (6), reliability (5), sensitivity/specificity (4), and misuse of the test (3).

A medical provider pointed out that this type of test may have uncertainties about whether the detected resistant bacteria are clinically relevant or merely contamination, making it less reliable.

The interviewee then went on to address a concern related to antibiotics overuse:

“I think overuse of antibiotics, again, a global issue, is such a huge problem. I just worry that taking this approach, letting potential users decide which antibiotics they should have, is problematic because, by default, and this is true even for physicians, people tend to think that broader is better. That’s not true. But you know that it’s really hard to get the message across that broader is not better.” (Int. 1-medical).

Another interviewee expressed concerns about whether people with positive test results would still consult a medical doctor.

“ for me probably the key question is what kind of bacteria it will indicate? And if, for example, it turns out that there might be some serious health issues. Then, I’m not sure that people are going to see medical doctors, and that’s my big fear, that they fail to follow up on care, either due to lack of resources or some kind of false information,” (Int. 11-expert).

### 3.3 Reliability, accuracy, and specificity of the proposed test

#### 3.3.1 Would you have any concerns about the reliability of the test?

In response to the question of reliability, various concepts were discussed, including accuracy (13), sensitivity/specificity (9), comparison to current lab practices (6), and correct specimen use (2).

Six respondents compared it to current tests on the market, like COVID-19 rapid tests.

“ It also depends on, like for Covid, our government has released some approved tests, and they were giving some percentage like, it’s 99% accurate, 98% accurate. So if it has government approval and like if they have released some statements, and this is reliable, I might trust it more,” (Int. 17-potential user).

Medical providers, experts, and potential users were keen to see how it compared to the current clinical tests used to detect antibiotic-resistant bacteria.

“Yes, I will have a concern about the reliability of this test. The one concern I have is how sure are we that it will give a proper diagnosis? How reliable will the test findings be? For instance, if you go to the lab … when they do culturing microorganisms, there are some environmental factors or abiotic factors, like even the temperature, can influence the results … These factors might affect the findings of the tests” (Int. 9-potential user).

#### 3.3.2 Reliability

Beyond the discussion generated by the specific question about the test’s reliability, twenty-five individuals discussed the concept of reliability. This included nine medical providers, six ethicists, and ten potential users. Medical professionals in particular mentioned the reliability of the tests in several contexts throughout their interview, including issues that might arise in the use of home tests, what potential users need to know about the tests, using the test directly with their potential users in the clinic, and what medical providers need to know specifically about the test.

The issue of reliability arose specifically in response to the following question (beyond the specific question about reliability): What do medical professionals need to know about the test? (13), concerns about the test? (10), if medical providers would feel comfortable using the test with potential users (3), if the test would decrease health disparities (2), the benefits of the test (2), and issues around home testing (2).

“I think my main concern is just how reliable the test would be if the test were really the same as sending it to the lab. Obviously, that would be ideal. But you know, point of care tests typically aren’t, so that’s my main concern” (Int. 8 -medical).

#### 3.3.3 Accuracy

The term ‘accuracy’ appeared 45 times across 22 interviews, encompassing transcripts from seven medical providers, six experts, and nine potential users. Discussions about the accuracy of the proposed tests arose 27 times when discussing the reliability of the test, either as an answer to a question about the test’s reliability or when addressing concerns the interviewee might have about the test in general.

Issues of accuracy arose seven times when interviewees responded to the question about what medical providers need to know about the proposed test.

“I would like once again, point to the accuracy and the precision of it, and probably if your provider understands the instructions for the test. Oh, if you like, you swab your cheek too hard, or you swab the wrong area. You might get a false positive or something similar,” (int. 19-potential user).

A medical provider stressed the relevance of accuracy as compared to speed:

“I guess I would be more interested in accuracy than speed, and in a lot of cases … we can just start broad and taper down to something narrower if we need to, if it’s accompanied by a symptomatic urinary tract infection, or something similar. Then we do want to make sure we’re treating the right thing, and we’re treating it appropriately,” (Int. 2-medical).

#### 3.3.4 Specificity and sensitivity

The term “specificity” came up 47 times and was mentioned in 18 different interviews, predominantly by medical providers (10), as well as by four ethics experts and four potential users. The term was most frequently mentioned in questions regarding concerns about the test (12), the test’s reliability (9), what medical providers need to know about the test (6), and the test’s benefits (3).

“So I think that I have like two major concerns. The first one would be that depending on the sensitivity and the specificity of the test, there might be false negative and false positive results, because the sensitivity and specificity is never 100%,” (Int. 25-medical).

Another medical provider pointed out that, in view of issues concerning the specificity and sensitivity of tests like these, often a second, more elaborate test needs to be done to confirm the diagnosis:

“I would rather be concerned about how specific and how sensitive these tests are. So that’s something that I think is a concern with almost all [tests] … But that’s gonna be more concerning because they’re not gold standard … You have to repeat the test … to confirm the diagnosis or confirm what’s happening,” (Int. 29-medical).

### 3.4 Affordability, accessibility, and health disparities,

#### 3.4.1 Do you think such a test would be easily accessible? Could this type of test help decrease health disparities?

Principal codes that appeared in this section include accessibility (17), affordability/decreased healthcare costs (9), comparison to current lab practices/tests (7), justice (3), possible consequences of the test (3), follow-up care/action needed (3), and cross-cultural comparisons (3).

Concerning health disparities, interviewees emphasized that the test itself must be affordable and that people must have access to medical doctors.

“It will help. But then, if it is not well priced, if the price is not properly regulated, the inequality gap may also come to play. Okay, whereby the rich are able to afford it while the poor are not,” (Int. 9-potential user).

“I think it … probably would be easily accessible. But health disparities are probably not based on people not knowing whether they are resistant to certain antibiotics or not. But it’s more about access to doctors themselves, access to hospitals, so I don’t know if it would decrease disparities.” (Int. 25-medical).

One expert commented on the nature of innovations, noting that they often demand a higher price tag and the need for some kind of regulations to keep prices reasonable.

#### 3.4.2 Accessibility

Beyond the answers to the above question which explicitly addressed accessibility, this code was mentioned 34 times in 21 interviews (comprising five medical providers, four ethics experts, and 12 potential users). It most often appeared in answers to the questions “What are the benefits of this test?” (6), “How much are you willing to pay for the test?” (5), and “Who should have access to rapid tests?” (3). The other codes that occurred along with “accessibility” included affordability/decreased healthcare costs (10), home testing (10), accuracy (9), convenience (9), rapidity of test (5), potential user guidance/what potential users need to know about using test (5), test interpretation (4) comparison to current lab practices/tests (4), possible consequences of test (4).

Interviewees emphasized that such a rapid test would offer accessibility advantages.

“With the explanation you gave, so far, I think it would be easily accessible. You can imagine a doctor coming to your house to conduct a test, and within 24 hours. Boom! The result is out. That’s accessibility” (Int. 5-potential user)

#### 3.4.3 How long would you be willing to wait for test results?

The question, “How long would you be willing to wait for test results” was only asked of potential users and experts, and fourteen individuals provided answers to the question. Answers ranged from immediate to 5 minutes (4), 30 minutes to an hour (3), by the end of the day (3), 24 hours (3), and a week (1).

“I know with these rapid test results, like COVID, you wait like 15 min and so I think people are accustomed to that. They want to know quickly. So I think that would be a good timeframe to shoot for” (Int. 15-potential user).

#### 3.4.4 How much would you be willing to pay for this type of test?

In this section, interviewees were asked two questions: first, whether they would be willing to pay for the test, and second, if so, how much they would be willing to pay out of pocket for the test.

Twenty-one individuals spoke about their willingness to pay out of pocket for the test, with 13 answering yes, four answering no, and four answering maybe. The main reasons individuals would be willing to pay include receiving a more rapid response, better overall healthcare, and being willing to pay if it were affordable. Almost all the individuals who answered ‘no’ had experience living in countries where healthcare was free. The four key factors included the overall health of the potential user taking the test, whether the test was necessary or would yield significant benefits to overall health outcomes, the cost of the test, and the test’s reliability.

In general, respondents were willing to pay for the test, with 15 willing to pay $10-$50, 9 willing to pay $50-$100, and 1 individual willing to pay $100-$200. Six answered other, citing that either healthcare should be free, the testing fee should be covered by insurance, or that they were unsure about the question.

“It depends on the answers to the first three questions. You know what the benefit is that I’m gonna have? And the accuracy and the reliability of the test, and the privacy issues. So, whether I’d be willing to pay much for it is a question. But I think that if I were willing to pay for this kind of test, it would be at the lower end of the range” (Int. 12-expert) (**Table 2**).

**Table 2:** How much would you be willing to pay for the proposed test.

How much willing to pay?	# of respondents
$10-50	15
$50-100	9
$100-200	1
Other	6 Responses included: Nothing, should be paid by insurance, price should be kept minimal, Unsure, Live in country with free medical care

Respondents who had experience living in countries that provide free medical care (France, South Korea, Germany, Netherlands) were either hesitant or unwilling to pay any money out of pocket for the tests.

Thirty-one individuals provided answers on how much they were willing to pay for the test.

#### 3.4.5 Affordability/decreased healthcare costs

Beyond the questions of “are you willing to pay?” and “how much are you willing to pay?”, nineteen individuals discussed issues of affordability/decreased healthcare costs. Of these, 10 were medical practitioners, 3 were potential users, and six were ethicists.

This code most often arose in connection with questions about the benefits of the test (9), whether the test would decrease health disparities (8), concerns about the test (3), and what medical providers need to know about the test (3).

“[It] is also going to help reduce the cost. Let’s look at it this way. You have the test material within your reach in your house, so you wouldn’t need to pay transport fare. So at least there’s a cost of transportation reduced for you” (Int. 9-potential user).

### 3.5 Test location

#### 3.5.1 Would you feel comfortable taking an at-home version of this test?

In general, potential users were the most positive about in-home testing. Eleven users stated they felt comfortable taking the test at home, one said it might be possible, depending on how invasive the test was, and three stated that they would feel more comfortable taking the test in a clinic. “Yes, cause I, you know, I’ve had experience with the at-home pregnancy test, and of course, at-home Covid tests” (Int. 15-potential user).

Ethicists were also generally positive about taking a home version of the test, with four giving unqualified yeses and three mentioning that they would probably feel comfortable, “…if it is an easy procedure that’s not possible to mess up…” (Int. 13-ethicist).

Medical providers were generally not comfortable with potential users taking a test of this type at home, with six saying no and five mentioning that it might be okay. They were concerned about issues such as antibiotic overuse, obtaining a correct sample, conducting the test correctly, and the need for effective communication to ensure proper treatment.

“There’s a big depends there. You know, I’m not really sure how you would … [Unlike] a COVID home test, there’s so many different types of bacteria, so many different resistance mechanisms, so many different types of antibiotics or classes of antibiotics. I don’t know what this test would look like [and] how that specimen is procured is going to be a little bit different for different types of infections, like, you know, if it’s somebody that’s worried about a skin or soft tissue infection. If it’s cellulitis. There’s no easy way to get a sample for that. You can’t just swab somebody’s skin, because that’s not necessarily gonna be the same organism that’s causing the infection” (Int. 2-medical).

#### 3.5.2 Home testing

Beyond this question, home testing was a significant subject in the interviews, mentioned 47 times across 19 interviews, with 7 medical providers, 4 experts, and 8 potential users talking about home testing, both as a benefit of the test, and as a possible concern. Outside of the question specifically about home testing, interviewees talked about this aspect of the test in answer to the benefits of the test (5), concerns about the test (7), only using the test in a clinical setting (6), the support needed to interpret test results (6), and the question about sexually transmitted infections (STIs) (4).

Medical providers and experts were excited about the possibility of a home test for STIs. Still, they were far more reticent about using home tests for antibacterial resistance, as mentioned above. Potential users discussed home tests as an exciting possibility and one of the benefits of the test, drawing comparisons to currently available home tests for COVID-19, but also citing the need for clear instructions on how to interpret the results.

#### 3.5.3 Do you think such a test should only be taken at a clinic or under medical supervision?

In response to this question, seven medical providers believed that this type of test should only be administered in a clinic or under the supervision of a medical professional. Two medical providers replied that it depends on how complicated the specimen connection might be. “I think it depends on the sample that you’re trying to take. Blood and some of the other more specialized samples that require a little more skill would be better done in a clinic, but some would be easier to do at home, right?” (Int. 32-medical).

Three experts also believed that the location where the test should be administered depended on the user’s ability to interpret the test results and agreed that it should only be conducted in a lab.

Of the potential users who responded to this question, three replied yes, it should only be taken under medical supervision, one was perfectly comfortable taking the test at home, and 10 again replied that it depends on the circumstances. Reasons given echoed those cited by medical providers and experts, who also stated that it depends on various factors, including the type of infection, difficulties obtaining the sample, difficulties interpreting test results, whether a person felt comfortable conducting a home test, and the age or overall health of the potential user.

### 3.6 What kind of advice or support would you need to interpret the test?

All interviewees answered this question. The principal codes that appeared were potential user guidance/what potential users need to know about using the test (13), test interpretation (13), comparison to current lab practices (7), home testing (7), follow up care/action needed, concerns/risks (4), need for public education (4), and accessibility (3).

Comparison to current lab practices and tests arose numerous times. Potential users commented on the need for clear instructions on how to administer the test, as well as an easy-to-read, pictorial indicator of the test results, like those used for a pregnancy or COVID test. Some suggested that a phone hotline or website with more information about using the home test would also be helpful. Medical providers were concerned about ensuring that potential users understood the risks associated with relying on this type of test, the reliability of the test compared to clinical tests, and providing clear instructions on how to use the test, as well as directions on what to do if a positive result is received.

Some medical professionals (2) commented that no support would make home tests of this kind possible.

“Yeah. At this point, I would say not to. Just based on what you know about it … I’d need to see a lot more detail about how the test works and what the sort of pitfalls of it are, and how it’s administered, and then I think I’d be able to [give better advice] to my patients of how they should be interpreting these results and how they should be administering the test if it’s one that they were administering home” (Int 2-medical).

### 3.7 How do you feel about potential users sharing their test results/sharing your test results via a web portal or a smartphone app?

In general, almost all interviewees felt comfortable sharing test results via a web portal or smartphone app, provided the system is secure and the information is kept private. Only two individuals said they would not wish to share their information that way, though in that case, they said it would depend on what information was being shared.

“Well, if my privacy is assured, I would feel no worry or no problem at all, but if my privacy is not assured, I’m gonna feel that I’m being betrayed because I am sending out sensitive information to an individual” (Int. 22-potential user).

### 3.8 Would any of your above answers change depending on what was being tested for, such as STIs?

In answering the question about sexually transmitted infections (STIs), the principal codes cited include privacy (9), possible consequences of test (6), confidentiality (5), shame/stigma (4), home testing (4), cross-cultural comparison (3).

Eight individuals did not perceive a significant difference between home testing for STIs and testing for antibiotic-resistant bacteria. Potential users (5) did not seem overly concerned about the difference, and two medical providers and one expert who responded similarly were equally skeptical about the safety of both types of home tests.

Of the twenty-four individuals who observed differences between an STI test and an antibiotic-resistant bacteria test, the main differences included the implications of both positive and negative results. Several respondents cited the hesitancy some individuals experience when accessing testing and healthcare for STIs and saw the possibility of home testing as a considerable benefit for the privacy and confidentiality of potential users. However, others saw issues, including sharing test results via an internet portal due to the sensitivity of STI diagnoses, worries about potential users misinterpreting results and overreacting, and possible user stress depending on the results received.

Others stressed the benefit of home testing for STIs.

“The huge benefit of using this for STIs is if the potential user is testing themselves, then they’re obviously either having symptoms or they’re wanting to protect their partner or whatever I think you know you’re making that anything that makes that easier prevents the transmission of these diseases. So I think that would be a huge benefit” (Int. 1-medical).

Other medical providers thought a home test for STIs would be more acceptable than one for ABR: “I would be more laid back if patients were testing themselves for STIs versus antibiotic resistance,” (Int. 30-medical). Still others felt less comfortable, as the market for STI home tests would be huge. The interviewee shared concerns about the misinterpretation of test results, the stress caused by receiving test results, and potential users not seeking the correct medical treatment following test results (Int 8-medical).

### 3.9 What do medical providers need to know about a lab-in-a-vial type test from your perspective?

Potential users and experts were explicitly asked, “What do medical providers need to know about a lab-in-a-vial type test from your perspective?” Answers varied, with the main answers focusing on reliability (9), accuracy (5), convenience (5), sensitivity (3), safety (3), need for public education (3), and training to administer the test (3).

One potential user stressed medical innovation:

“In my perspective, medical providers should be made to understand that there is a need for innovation, flexibility, convenience, and creativity in medical practice, even when there is also the need to ensure that the codes of medical practice are followed. But they should also understand that there are times when we have to embrace a new approach to things” (Int. 9-potential user).

Medical providers were asked the same question, and spoke of sensitivity/specificity (8), reliability (4), specificity (4), affordability (3), and the possible consequences of test results (2).

“What is the evidence? What are the chances that you test a false positive or a false negative to inform the potential users? And then also I would think it would be great to have attached certain rules like if you have a potential user test positive, then it also means something, you don’t just test positive but then don’t have a treatment for the potential user” (Int. 23-medical).

### 3.10 Medical provider questions

#### 3.10.1 Please briefly describe your place of work and the potential users you work with.

The eleven medical providers we interviewed provided descriptions of their workplaces and the potential users they served. The medical providers we interviewed included six individuals from the United States, one individual from Pakistan, and four Germans. Six medical providers were female, and five were male. There was a wide diversity in specialization, ranging from pediatric and adult infectious diseases, pediatric oncology, research on antibiotic overuse, to working as a general practitioner in both urban and rural practices. One interviewee was finishing a medical residency in internal medicine, and still another worked at the U.S. Department of Veterans Affairs. The patients they served were equally diverse, from all age groups and from a variety of backgrounds.

#### 3.10.2 How comfortable would you feel using this kind of rapid results test with your potential users?

Only medical professionals were asked this question, and all answered. Codes that showed up most include accuracy (3), reliability (3), comparison to current lab practices (3), and rapidity of test (2).

Three medical providers answered with a qualified yes, citing their experience with rapid tests, that they would stop if potential users experienced any adverse side effects, and assuming that the test’s accuracy, reliability, and specificity are verified.

“Yeah, I mean, I think if it was FDA approved, and if it was covered by insurance, and if it was … as good or almost as good as the lab test. I think that would make sense. I think … initially, we would you know, do the rapid [test]. And if it’s negative, then maybe send something to the lab, anyway. So that’s kind of the other factor, it may not eliminate all of our testing, but it has the potential to give us an answer faster” (Int. 8-medical).

Eight answered with a maybe, citing concerns about the specificity and reliability of the tests, the use of these tests with immunocompromised individuals, the type of infection being treated, and again emphasizing the need for evidence-based medicine.

#### 3.10.3 Who should have access to this kind of test?

Of the medical providers who answered this question, six thought the rapid test should be limited to those able to prescribe antibiotics, and two believed the answer depended highly on the type of antibiotic-resistant bacteria being tested for. Two believed everyone should have access to the test.

“Yeah. and I think that it just depends on how broad you want to distribute it? Because I think the big question is, should it be administered in a clinic? And if so, you know, the only real advantage is its speed, but speed is very helpful. So, I think that would be an improvement if you can say that it’s accurate. If it’s faster, it’s better. But you know the big question is, if you’re gonna give it to someone at home and can ensure that it’s appropriately done, that’s a game changer … So if you can do it at home and transmit those results, you know, safely and accurately, then that’s the ideal”. (Int. 31-medical).

One medical provider reflected on testing the water supply and sewage water in hospitals:

“[But] if you could, for example, test water supplies for hospitals, it could make a big difference to see if they have antibiotic resistant bacteria in their water supply, or also in their wastewater to then implement filter systems or anything” (Int. 23-medical).

#### 3.10.4 What kind of advice do you have for our research group in developing this kind of test?

Six medical providers responded to the question, “Do you have any advice for our research group?” Advice ranged from focusing on specific types of antibiotics, especially the most commonly resistant ones.

“So, I think I would start with a test that focuses on certain antibiotics and certain types of resistance and then try to test it in, like, with these specifications in certain settings, for example, if you have like pneumonia, and you know that Streptococcus pneumonia is the most common pathogen causing this disease. And you could try to develop a test specific for this situation and test it in this specific situation” (Int. 25-medical).

Other interviewees offered additional insights into the test based on the interview questions. Medical professionals were generally wary of the potential benefits of the test.

“It can be helpful. [But] it seems like only one tool in a large number of [tools needed]. And not only that, but this is the most necessary point in developing such tests that the context must be absolutely clear. How to use it? Who is using it? And for what? And what are the consequences?” (Int. 21-medical).

Other medical providers and experts were excited about the potential use of this kind of test insofar as it promotes access to effective antibiotics and lowers the cost for potential users.

### 3.11 High count topics

#### 3.11.1 Key aspects of the test

##### 3.11.1.1 Convenience

Nineteen interviewees commented about the convenience of the proposed test: nine potential users, four experts, and six medical practitioners. This aspect of the test was most often mentioned as part of answers to questions about the benefits of the test (9), what medical providers need to know about the test (4), home testing (4), sharing results of the test online (4), and how much individuals are willing to pay for the test (3). Some interviewees mentioned issues of convenience four to five times in response to different questions throughout their interview, and convenience was raised in relation to 13 other questions overall.

Most potential users and experts were excited about the convenience of the test, viewing it as a significant benefit and potential cost-saving for them. Doctors who responded were concerned about compromising the overall accuracy of the test for the sake of cost.

“…it could be convenient, but what are you losing for that convenience? When I say convenience, I should also add, you know, the cost of it, because in places like Pakistan, sometimes potential users will already know about these tests, and then will get upset at you for sending the gold standard test because it’s more expensive, and these kinds of tests are usually much cheaper. There is this risk that you always take by not doing the better gold standard testing” (Int. 1-medical).

##### 3.11.1.2 Rapidity of test

Discussions about the test’s speed came up 45 times in 27 interviews (11 medical providers, 13 potential users, and three ethics experts). This attribute of the proposed test appeared most frequently in the question about its benefits (22) and less regularly when discussing how long individuals would wait for their results (4). The code appeared most frequently in connection with other codes, such as convenience (13), accessibility (9), early detection (9), home testing (8), affordability (8), and comparison to current lab practices/tests (7).

Medical providers focused on how rapid tests can improve the treatment offered to potential users and reduce antibiotic overuse.

“I think, especially if the goal is to develop a test that can identify antibiotic resistant bacteria. The advantage of that is tailoring antibiotics, so oftentimes we’re sort of unsure whether we know we make these guesses right about whether we need mercury coverage or those kinds of things, and I think it could cut down on antibiotic overuse, if we can tailor our treatment” (Int. 8-medical).

Potential users and experts focused more frequently on the personal benefits of the test’s rapidity, including lower costs, easier access, and reduced waiting time.

“My own understanding, [is] that number one is going to save time. You don’t necessarily need to go to the lab or wait for lab results to come out before, so the doctor … does the test in his office, gets the results, and boom, he starts his treatment. So I think it’s going to save time and stress, too” (Int. 5-potential user).

#### 3.11.2 Proper use of test

##### 3.11.2.1 Potential user guidance/what potential users need to know about using the test

The code “potential user guidance/what potential users need to know about using test” appeared 38 times across 20 interviews (8 medical providers, 3 experts, and 9 potential users). The code appeared most frequently in questions about the type of support potential users might need to interpret test results (13), whether the test should be used only in a clinical setting (5), and whether individuals should be able to use a home test (4). Other codes that appeared with this code include home testing (14), test interpretation (14), possible consequences of test (11), need for public education (10), accessibility (10), follow-up care/action needed (6).

“I think one of the disadvantages is getting people to know how to use the test kits … [because] it is a new way of running a test. Getting people to get trained and get the reliable information on how to get this test done, especially the medical practitioners, is actually something of concern” (Int. 22-potential user).

“I think the kind of support that you’d have to provide to potential users would be making sure that they understand the limitations of the test. The benefits of the test, too, I would imagine, for example, such as STI screening. I don’t think we’re testing for antibiotic resistance often or ever, really,” (Int. 8-medical).

##### 3.11.2.2 Possible consequences of the test

Discussion of the possible consequences of the test occurred 43 times across 14 interviews. Of these, six were medical providers, five were experts, and three were potential users. This theme came up most often when discussing support for interpreting test results (5), STI testing (5), concerns about the test (5), and home testing (4).

When discussing concerns about the ABR test and home testing, worries included the overuse of antibiotics, misuse of the test, and potential stress from the test results.

“So if I knew about the like properties of the test, then I would certainly give it a try. But if I experience patients becoming overly anxious, or maybe, if in my experience, the outcome for the patient was negative, I would stop, but I would definitely give it a try,” (Int. 25-medical).

##### 3.11.2.3 Test interpretation

Discussion of test interpretation appeared 41 times in 20 interviews (6 medical providers, 4 ethics experts, 10 potential users). The code appeared most often in discussions about the support potential users might need to interpret the test results (13), and less frequently in questions regarding home testing (5), concerns about the test (4), and the benefits of the test (3).

“I’d rather potential users did it in the office, generally, because okay. Number one, if you take that home test. Did they administer it properly?… Number two is how do you know that they communicate with you effectively and get it treated?” (Int. 28-medical).

“I would definitely see the merit in that … I think that some people don’t fully grasp the idea of what antibiotic resistance is. So if you … have a panel, come back and it’s like, Oh, you’re resistant to staph aureus, I feel like somebody might not be able to be like, okay, I’m resistant to this, what does this mean? So I guess I would understand the benefit of having it at, like the healthcare provider, only just for the fact that, like, they might be able to interpret and explain the consequences of the results to somebody” (Int. 19-potential user).

#### 3.11.3 Comparison to current lab practices/tests

Interviewees drew comparisons to current lab practices or tests 66 times in 22 interviews (7 medical providers, 5 ethics experts, and 10 potential users). Comparisons to currently available tests and current lab practices showed up in answers to almost all of the questions asked, though most prominently in the questions about the use of home tests (8), support needed to interpret results (7), if the proposed test will decrease health disparities (7), how long are you willing to wait for results (6), benefits of test (5) sharing test results online (5).

Comparisons ranged from drawing on personal experiences using standard home tests for COVID-19 (by 15 interviewees) and pregnancy tests (5), to experiences involving visits to a doctor’s office for lab analysis of results and using online health portals to send and receive test results. Medical providers, ethics experts, and potential users drew on experiences with these kinds of tests to calibrate how long they would be willing to wait for test results, assess the benefits of the tests, consider concerns about their reliability and accuracy, and evaluate their willingness to share test results online.

“So I have experience with rapid tests via, of course, pregnancy tests, being a woman, and with COVID-19 tests. Right? And you know, just about all of us have experience with the COVID-19 test at the very least. And I think it’s highly convenient to be able to have access to a test that is fairly simple to perform, and provides accurate results within a matter of, you know, 15 to 20 min. So that you can make decisions. It’s economical, it’s convenient. It prevents one from having to go all the way into the doctor’s office, and it avoids complications with insurance” (Int. 15-potential user).

The discussion of commonly used home tests arose in the context of health disparities, specifically regarding how a home test might increase access to and affordability of appropriate antibiotics, similar to currently available home tests such as those for COVID-19 and glucose.

#### 3.11.4 Cross-cultural comparisons

We had several interviewees coming from countries outside the United States, as well as those who had worked or lived internationally. All these interviewees were able to make interesting cross-cultural comparisons about how this kind of test might work or fail in such diverse situations. Overall, nine interviewees—three medical, one expert, and five potential users —provided cross-cultural comparisons of this kind.

One medical provider (interview 1) spoke extensively about her experience working in a country in South Asia, where antibacterial resistance is extremely high and individuals can get antibiotics without a prescription. In discussing the potential benefits of the test, she first noted how it could be instrumental in the United States. Still, she then had this to say:

“In Pakistan, it’s a little bit different, because we already have such a high burden of disease and there is, antibiotics available over the counter. So, it gets really tricky in places like that, because interpretation of the test is difficult for the layman. I think the doctor’s office is one thing, but if this were like a home-based test, which would actually be really helpful if you had an educated population, but that’s not the ground reality. This kind of test without being able to interpret it especially in a community where you really need to conserve your antibiotics, it can have some dangers associated with it” (Int. 1-medical).

Potential users with an international background often drew comparisons between the types of support they received when reading test results and what they would be willing to pay for the test. They discussed how national healthcare systems, be it government-subsidized, private, or a mixture of the two, would have a significant influence on how much tests might cost and how available they might be, and how willing potential users would be to share test results in an online format (Interview 11, 3, 9, 5).

“I don’t think it’s best to pay for these tests, period. I don’t like the medical system in this country, anyway. France, you would never have to pay for [test results], if you’re sufficiently sick that they’re testing you for antibiotic resistant bacteria. Shouldn’t we have free medical care?” (Int. 3-potential user).

Participants living in South Asia and Africa often discussed the benefits of this kind of test. One person said:

“But I think it’s very beneficial in situations where we don’t have medications available. [Unlike the United States], other countries don’t always have those antibiotics available, especially when you go to the public sector, you know, governments run hospitals in other countries … So it’s gonna help over there that they will, you know, do with whatever they have at work. (Int. 29-medical).

#### 3.11.5 Antibiotics overuse

The topic of antibiotic overuse was discussed 16 times across eight interviews with six medical providers and two experts. No potential users brought up this topic.

Two interviewees discussed concerns about antibiotic overuse in relation to the test. One ethics expert addressed the potential tension if users believe the test is used to ration antibiotics, prioritizing the societal fight against overuse over individual treatment.

“Well, I can imagine that there could be some tension there between [is] the treatment being withheld because it really would not be effective? Or is the treatment being withheld for more social reasons?… People already complain that antibiotics are harder to get than they used to be because doctors are a little bit more conscious about overprescribing them. Might they mistakenly think that the test is designed to sort of ration antibiotics? (Int. 16-expert).”

Two medical providers and one expert discussed the benefits of this test in fighting antibiotic overuse:

“The thing is that when we get a patient and [they] get prescribed different kinds of antibiotics, and [are] resistant to different antibiotics. That rapid test is gonna make treatment faster, easier. Sometimes we go very broad spectrum when a patient comes into the hospital. Here, we could narrow it down [with] a rapid test” (Int. 29-medical).

## 4 Limitations

We have identified several limitations for this study. First, the interview sample was relatively small, and the interviewees volunteered to participate, making the sample self-selected. Interviewees chose to join for personal reasons, regardless of their views on the topic. This limits the generalizability of the findings. We attempted to mitigate this by interviewing a diverse group of stakeholders from various countries to capture a broad spectrum of responses and perspectives. Our study is also extremely over-representative in terms of education level, with 75% of participants holding a master’s degree or higher. This is somewhat accounted for by the fact that 56% of the interviewees were either an ethicist with an MA or above or a medical provider with an MD, but the potential user population was still more educated than the average population. Our recruitment method also likely attracted individuals who were already interested in technology, skewing our results toward greater technological optimism, especially among potential users.

Secondly, a limitation that likely affected the study population was the decision to conduct interviews via Zoom. While this choice had the benefit of automatically generating a transcript of the interview, it also required recipients to have access to a computer and a stable internet connection.

Thirdly, the lack of details in describing a hypothetical rapid test for ABR that could be done in a medical clinic or at home greatly limits the use of stakeholder feedback when addressing the specific application, utility, or implementation of such a test. However, we believe this feedback raises significant ethical questions and essential considerations when seeking technological solutions to antibiotic overuse.

Another limitation is our oversight in failing to provide clear, accessible definitions for some terms used in the interview protocol. In retrospect, it would have been beneficial to define “reliability.” As we did not provide this clarity, some survey respondents without a medical background seem to have been confused by the term. Finally, due to the nature of the research this ethics study was connected with, we were unable to specify the type of rapid test being developed, except that it was for antibiotic-resistant bacteria.

These limitations should be considered when interpreting the findings of this project.

## 5 Discussion

In analyzing the responses to our interview questions, several interesting issues emerged, including different perspectives among potential users, medical providers, and ethics experts, questions about in what context these kinds of ABR tests could be helpful, and cultural differences that might need to be considered when developing and using these kinds of tests. Given the hypothetical nature of the test described here and the very real concerns noted above about gathering uncontaminated biological samples for a home test, no real conclusions about the actual validity of this kind of test can be drawn from this study, as is commonly the case when discussing emerging technologies. Additionally, the small sample size of this study (32 individuals) and the fact that participants’ education skewed strongly toward advanced degrees mean that the discussion below should be taken in context. Nevertheless, we believe that the results of this study point to several key ethical issues to consider in the development of point-of-care tests.

Key differences between potential test users and medical providers and experts were perhaps the most apparent. When examining potential users of the test, the majority were relatively optimistic about the possibilities the ABR test could offer. A proportion of interviewees, medical providers, ethics experts, and potential users were enthusiastic and confident about the convenience and speed of the proposed test, with the caveat that it should be assumed to be accurate and reliable as conventional lab tests for ABR. This resonates with a study examining the use of tests for diagnosing acute respiratory tract infections, which identified rapidity and accuracy as key features for adopting this type of test (Hoste et al., 2023). Potential users also viewed such a test as potentially more affordable, depending on its price point, as it might save on travel and doctor’s office costs. These issues of accessibility and affordability were key in the ethical analysis of the case. Interviewees who cited these factors as valuable placed them in the context of making targeted antibiotic use more accessible to a broader segment of the population. Even if the actual feasibility of a direct-to-consumer ABR test is somewhat far-fetched, interviewees focus on accessibility and affordability as important traits of this type of test and point to the need to incorporate them into the development of future ABR tests. While the possibility of an at-home ABR test seems far from being developed, the enthusiasm among our potential users, grounded in assumptions of accessibility and affordability, underscores a focus on normative principles such as autonomy and justice. With unequal access to healthcare still prevalent in societies worldwide, patients are increasingly seeking ways to enhance control over their own health and to use alternative methods, e.g., telehealth or at-home tests that can be ordered online. When imagining the hypothetical test, potential users prioritized features usually associated with health justice, including affordability and accessibility, as well as ease of use and educational resources to ensure understanding.

Differences emerged again between potential users and medical providers/ethics experts, with potential users not being overly concerned about reliability and accuracy, or with issues of interpreting test results. Potential users assumed in our study of a hypothetical test that any rapid ABR test developed would likely be reliable and accurate. Similarly, in a study examining patient preferences for point-of-care testing, the authors found that most interviewees without a medical background believed these tests would enhance their overall medical care and had few concerns about interpreting test results. Instead, they valued POC tests for their rapidity and ease of use (Howard-Wilson et al., 2024). In contrast, medical providers and experts in this study were more cautious and were more likely to raise concerns. Some potential users noted that if the U.S. Food and Drug Administration or similar agencies in other countries approved the test, they would be happy to use it. This resonates with findings from developers of similar tests, who emphasize the need for tests used in less controlled environments by users with little to no medical training to be accurate in real-world conditions. Therefore, they must be robust and easy to use (Farmer et al., 2022).

The quality of the suggested test played an essential role in many interviews. Interviewees without a strong background in bioethics or medicine often did not clearly distinguish between the terms “reliability” (the consistency of measurement over time and under different conditions) and “accuracy” (the closeness of a measurement to the actual or accepted value of what is being measured). One reason might have been the failure to provide interviewees with a clear definition of reliability. Confabulating these two terms was common among potential test users, including individuals with higher degrees but no medical background. This points to the ethical issue of health literacy and the need to introduce concepts people may not be familiar with and provide definitions for the concepts. Similarly, discussion around the “reliability’ of tests abounded during the COVID-19 pandemic in the news and social media but rarely provided a discussion of what “reliability” actually means. These findings point to the ethical need to provide more public education around these issues as the field of rapid and at-home diagnostics continues to grow. Others discussed false positive or false negative results, implying that a “good” test will yield the correct result. Some interviewees with an apparent background in research or medicine used the terms “sensitivity” and “specificity”. Given this clear overlap, we present and discuss reliability, accuracy, sensitivity, and specificity together, treating them as aspects related to test accuracy.

Interviewees from both medical and non-medical backgrounds often drew comparisons with COVID-19 rapid tests when discussing reliability and accuracy, reflecting the frequent news coverage during the COVID-19 pandemic. It appears that the COVID-19 pandemic and the rapid at-home tests developed in this context influence interviewees’ views on rapid tests. Other studies looking at point of care tests have cited similar results (Hoste et al., 2023; Howard-Wilson et al., 2024). However, there are also several significant differences between COVID tests and the type of test discussed in the interview. It seems plausible to assume that a different kind of specimen would be required, making the test more challenging to apply and taking longer to obtain results. Thus, it seems that home tests for COVID-19 have made the topic appear less complex. Interviewees, especially potential users, may underestimate the difficulties and possible issues associated with this type of test. As noted by Farmer et al. (2022), “the uncontrolled environment and minimally trained user scenario that encompasses home use” poses several challenges to test validity (Farmer et al., 2022, 2). Even though potential users interviewed in this study underestimated the difficulties of developing an actual ABR rapid test for use in a home environment, their claims still point to important ethical insights. For instance, COVID-19 introduced the general population to another way to access healthcare. This way could allow a larger portion of the population to obtain the healthcare they need and could be a potential solution to addressing existing inequalities.

The same influence by COVID-19 tests may play a role in the often relatively positive perception that potential users of these tests expressed in their responses. This may change with a specification of the exact conditions for sample collection, including the time and procedure for receiving test results.

Medical providers and experts were more hesitant about the benefits of an ABR rapid test, especially for home use. Two interviewees expressed concerns about how the proposed ABR rapid test would hold up to comparisons with “gold standard” testing for antibiotic-resistant bacteria, and other medical providers repeatedly worried about patients’ ability to collect correct specimens for testing or to interpret test results. Similar to points raised by Farmer et al., 2022 who were focusing on factors critical to the development of point of care tests in general, questions raised by medical providers and expert interviewees in our study echo the importance of human factors in the context of home testing, including the test users’ ability (for example, low vision), the environment and context the test is being performed in (low light, high humidity), and the ability to interpret the test results (Farmer et al., 2022). Additionally, these stakeholders raised questions about the test’s actual use, noting that antibiotics in many countries require a doctor’s prescription. And, given the vast number of different bacteria present, how would a user select the correct test? Another primary concern was whether users of the test seek appropriate treatment after receiving a positive result. One interviewee thought that a test to determine if the bacteria causing a urinary tract infection are antibiotic-resistant might be helpful, but other medical providers remained skeptical.

Medical providers and ethics experts also provided valuable discussions about the potential role ABR rapid tests could play in medical practice. Some seemed more positive about point-of-care testing that could be done in a clinic or allowing patients to use a home test to provide initial results, followed by more standard lab testing. In contrast, if the ABR rapid test could serve as a substitute for lab tests, there would be high requirements for reliability and accuracy. Other medical providers discussed how these tests would be beneficial in hospital settings, where exposure to ABR is high; such tests might help guide targeted treatments for infections. One expert and one medical provider also mentioned the usefulness of ABR tests for testing hospital water and wastewater for ABR.

The overuse of antibiotics was a key issue among medical providers and experts. Two experts viewed this type of test as a potential tool in combating the overuse of broad-spectrum antibiotics. At the same time, one user was concerned that patients might resist the test if they believed it was being used to ration certain types of antibiotics. This fear of discrimination and distrust of healthcare providers, given the history of medicine, is understandable. This has led to arguments that rationing and resource allocation should be handled at the policy level, not at the bedside. This concern echoes findings from the Hoste et al., 2023 study, in which interviewees discussed how, even though a rapid test for acute respiratory infections could be used to rule out a bacterial infection, patients might insist on antibiotics regardless of the test results (Hoste et al., 2023). While ABR rapid tests that patients can perform at home may not be the solution, issues of health literacy, open, ongoing discussions about the problem of antibiotic overuse and its impact on individuals, as well as seeking solutions that empower individuals to be active partners in their health care may point the way forward. All of these may encourage a more sustainable use of antibiotics and help mitigate or overcome the existing tragedy of the commons.

In terms of home testing, experts and medical providers were more positive when answering the question about rapid home tests for sexually transmitted infections rather than ABR, citing issues of privacy, protecting sexual partners, and overall helping to prevent the spread of STIs. However, concerns about misinterpreting results and seeking correct medical treatment remained. This tendency to be more positive about rapid home tests for sexually transmitted diseases indirectly points to interviewees’ doubts or concerns about the suggested rapid test for ABR.

Regional and cultural aspects also play a role in the benefits of an ABR home test. Studies have found that in countries with limited surveillance of resistance development, clinical misuse of antibiotics tends to be more widespread, and where users and patients can freely access antibiotics, ABR levels tend to be higher (Chokshi et al., 2019). In these situations, a rapid test is more likely to be helpful. One interviewee in this study, a medical provider with experience working in a country in South Asia and the United States, mentioned this context.

Given the exploratory nature of the research project this interview study was connected to, the research team was unable to be overly specific about the kind of test being developed and posed interview questions about “antibiotic-resistant bacteria” without specifying what we meant, or if the test would be used in the context of a specific disease or bacteria. This issue was raised by many of the medical providers we interviewed.

Overall, many interviewees focused on the question of what additional benefits the proposed test type would provide.

Currently, existing tests on the market are primarily focused on clinical and point-of-care use in medical offices or similar settings, where a trained professional is available to administer the test and interpret the results. While current advances in ABR testing seem able to provide rapid and robust results for antibiotic surveillance, or letting hospitals or clinics know what ABR bacteria are present in a given facility, the question remains whether a rapid test like the one proposed could potentially provide some support to prescribers who are trying to quickly treat an infection with a targeted therapy or limit the off-target use of antibiotics by prescribers afraid of being accused of withholding broad-spectrum treatment by patients?

Alternatively, how would such a test empower potential users? In a previous publication from a different section of the same interview study (Laas et al., 2025), interviewees cited the potential empowerment that self-administered tests for environmental health-related factors might provide individuals, enabling them to become more aware and active in managing their health. Interviewees also viewed environment-related tests as enabling communities to collaborate based on test results to improve environmental factors in their lived environment, such as air pollution. In a study examining point-of-care tests for managing chronic illnesses, potential users were generally positive about how these tests could assist in diagnosing and managing their health (Howard-Wilson et al., 2024). Self-administered ABR tests are unlikely to be the best example of these tests, given real concerns about antibiotic overuse. However, these tests have the potential to reduce antibiotic use. In addition, this type of test could help patients become more educated about the dangers of ABR and increase knowledge of how prevalent ABR is in their own bodies and even communities.

## 6 Conclusion

While this study highlights key ethical aspects to consider for a hypothetical, rapid, point-of-care test for antibiotic resistance, it can only offer general guidance on potential benefits, risks, and specific areas that need more attention. Findings from this study point to the key relevance of accessibility and affordability when developing diagnostics of this kind, as well as the overarching need for health literacy around antibiotic resistance. This includes developing a shared understanding of what is meant by key terms when discussing these kinds of diagnostics, so both patients and medical providers are speaking the same language. While prospective users of this type of hypothetical test were generally positive about the proposed test, this might reflect public acceptance of point-of-care tests in general, rather than one specifically measuring ABR. Medical providers and experts knowledgeable about the problems of antibiotic overuse quickly identified drawbacks and areas of concern with home testing for ABR, offering helpful guidance on where further research and consideration are needed.

Given the exploratory nature of this study, we were unable to specify the type of test we asked stakeholders about. Future interview studies of this kind should be more specific and include a detailed scenario describing a test specific to one type of bacteria. This developed scenario would elicit more detailed feedback on its usefulness in particular settings and the possibility of use in a home setting, given the difficulties associated with the type of specimen that must be collected, the test price, and related aspects.

Given the worldwide growth of antibiotic resistance, there is a real need for advances in diagnostics to help practitioners and the public be better stewards of antibiotics. A recent report from the World Health Organization found that in 2023, approximately one in six bacterial infections worldwide were caused by antibiotic-resistant bacteria. In Southeast Asia and Eastern Mediterranean countries, this reached one in three (World Health Organization, 2025, p. 9). A test like the hypothetical one discussed in this study could be helpful if it included many of the considerations and concerns mentioned above. However, more research is needed, especially when more specific information about such a type of test is available.

## Data Availability

The raw data supporting the conclusions of this article will be made available by the authors, without undue reservation.
