# Supplementary material for: Stakeholder perspectives on a hypothetical rapid test for antibiotic resistant bacteria: an exploratory study
Source: Front Antibiot. 2026 Jan 12;4:1729093. doi: 10.3389/frabi.2025.1729093 (PMC12833287; doi:10.3389/frabi.2025.1729093)
Supplement: Supplementary file 1 [file DataSheet1.docx]

**Stakeholder Perspectives on a Rapid Point of Care Test for Antibiotic Resistant Bacteria**

# **Appendix I: Surveys**

**Potential Test User Questionnaire**

Demographics

1. What gender do you identify as?
2. Male
3. Female
4. Open Answer
5. Prefer not to say

2. What is your age?

1. 18-30 years
2. 30-45 years
3. 45-65 years
4. 65 +
5. Prefer not to say.

3. Please specify your ethnicity.

1. African-American or Black
2. Asian
3. Caucasian
4. Latino or Hispanic
5. Native American
6. Native Hawaiian or other Pacific Islander
7. From multiple races
8. Prefer not to say
9. Open answer

4. What is your nationality?

5. What is the highest level of school you have completed or the highest degree you have received?

1. Less than high school degree
2. High school degree or equivalent (e.g., GED)
3. Some college but no degree
4. Associate degree
5. Bachelor's degree
6. Graduate degree

6. How often do you visit a healthcare provider?

1. Once a month or more
2. Once every three month
3. Once a year or less
4. Other (space for short answer)

7. Do you have any chronic health conditions?

1. Yes
2. No

We are working with a team of researchers to develop a method of swiftly detecting antibiotic-resistant bacteria or other similar infections without sending biological samples to a lab. This project is currently at an early stage, and while we do not have details of how the test will actually work, this study is meant to help our team better understand some of the major ethical concerns potential users and medical providers might have in using the test and hopefully address many of these issues in its development. Current methods for detecting antibiotic-resistant bacteria can take 24-48 hours under the best circumstances. The goal is to develop a test that can detect antibiotic-resistant bacteria quickly. This should allow potential users to get treatment quickly rather than waiting for lab results to return.

The proposed test could be either at a doctor’s office or health clinic or potentially even administered at home by the potential user, somewhat like a COVID test.

The goal of this interview is to gain some insight into the benefits this type of test might have for potential users and what questions or concerns potential users might have with this type of test.

1. What do you think might be the benefits of using a test like the one proposed?

1. What concerns would you have in using a test like the one proposed here?
2. Would you have any concerns about the reliability of the test?
3. Would you be willing to pay for the immediate results available via this test versus the more traditional test that takes longer to get results?

1. How much would you be willing to pay for this type of test?
2. $10-50
3. $50-100
4. $100-200
5. $300-500
6. $500+
7. Do you think such a test would be easily accessible? Could this type of test help decrease unequal access to healthcare?
8. Would you be comfortable taking this test at your doctor’s office or a health clinic? (So, there is no need to send samples to a lab)?
9. Would you feel comfortable taking an at-home version of this test?
10. What kind of support would you like to have to interpret the test results?
11. How long would you be willing to wait for the test results?
12. How do you feel about sharing test results on a smartphone with your healthcare provider?
13. Do you think such a test should only be taken at a clinic or under medical supervision?

13. Would any of your previous answers change depending on what was being tested for, such as sexually transmitted infections?

14. What do medical providers need to know about this type of test from your perspective?

**Medical Provider Questionnaire**

Same demographic information as asked patients, with the following question added:

1. Please briefly describe your place of work and the patients you work with.

We are working with a team of researchers to develop a method of swiftly detecting antibiotic-resistant bacteria or other similar infections without sending biological samples to a lab. Current methods for detecting antibiotic-resistant bacteria can take 24-48 hours under the best circumstances. The goal is to develop a test that can detect the presence of antibiotic-resistant bacteria quickly so that effective treatment can immediately be given.  The proposed test could be either at a doctor’s office or health clinic or potentially even administered at home by the patient, somewhat similar to a COVID test. The goal of this interview is to gain some insight into the benefits this type of test might have for patients and what questions or concerns patients might have with this type of test.

1. What do you think might be the benefits of using a test like the one proposed?
2. What concerns would you have in using a test like the one proposed here?
3. Would you have any concerns about the reliability of the test?
4. How comfortable would you feel using this kind of rapid-results test with your patients?

1. Do you think such a test would be easily accessible? Could this type of test help decrease health disparities?

1. Would you feel comfortable with your patients taking an at-home version of this test?
2. How do you feel about patients sharing test results with you via a web portal or a smartphone app?
3. Would this change depending on what was being tested for, such as STIs?
4. What do medical providers need to know about a lab-in-a-vial type test from your perspective?
5. Do you think such a test should be taken at a clinic or under medical supervision?
6. What kind of advice or support would you need to provide your patients about using this kind of test?
7. Who should have access to this kind of test?
8. What kind of advice do you have for our research group in developing this kind of test?

# **Appendix II: First Level of Coding-Full Interview Transcript**

| **Code** | **# mentions** |
| --- | --- |
| Accessibility | 5 |
| Accuracy | 2 |
| Advice for Research Group | 1 |
| Affordability/Decreased Healthcare Costs | 2 |
| Antibiotic Overuse | 17 |
| Anxiety/Stress of waiting for test | 7 |
| Benefits | 23 |
| Change in medical practice | 12 |
| Comparison to Current Lab Practice/Tests | 72 |
| Concerns/Risks | 36 |
| Confidentiality | 16 |
| Convenience | 42 |
| Correct Specimen Use | 16 |
| Cross-Cultural Comparison | 42 |
| Decrease mortality | 4 |
| Description of Potential users | 6 |
| Diagnosis and Use | 13 |
| Diverse Medical Situations | 13 |
| Early Detection | 21 |
| Environmental Concerns | 8 |
| Finding Correct Antibiotic | 17 |
| Follow up care/action needed | 27 |
| Health Disparities | 16 |
| Home Testing | 50 |
| Justice | 10 |
| Lab test needed as followup | 6 |
| Misuse of test | 13 |
| Need for Public Education | 24 |
| Optimizing healthcare | 7 |
| Potential user autonomy | 5 |
| Potential user Guidance/what potential users need to know about using test | 38 |
| Point of Care Testing | 4 |
| Possible Consequences of Test | 51 |
| Privacy | 29 |
| Rapidity of Test | 49 |
| Reliability | 55 |
| Restricting Rapid Test to Clinic | 10 |
| Role of physician | 20 |
| Safety | 9 |
| Sensitivity/Specificity | 47 |
| Shame/Stigma | 6 |
| Technology Adoption | 24 |
| Test Interpretation | 42 |
| Thoughts on Administering Test to Potential users | 4 |
| Thoughts on introducing/marketing a new test | 8 |
| Training to administer test | 15 |
| Trust/Transparency | 2 |
| withdrawal from medical consultation/ self care | 7 |
|  |  |

**Appendix III Participant Demographics**

| **Characteristics** | **Number** | **Percentage** |
| --- | --- | --- |
| **Interviewee Category** |  |  |
| Medical Provider | 11 | 34% |
| Ethicist | 7 | 22% |
| Potential UserPatient | 14 | 44% |
| **Gender** |  |  |
| Male | 15 | 47% |
| Female | 17 | 53% |
| **Ethnicity** |  |  |
| Caucasian | 14 | 44% |
| African/African American | 7 | 22% |
| Asian/South Asian | 8 | 25% |
| Hispanic | 1 | 3% |
| Multiple Ethnicities | 2 | 6% |
| **Age** |  |  |
| 18-30 | 10 | 31% |
| 31-45 | 11 | 34% |
| 46-65 | 8 | 25% |
| 65+ | 3 | 9% |
| **Education Level Achieved** |  |  |
| High School | 2 | 6% |
| BA | 6 | 19% |
| Masters | 9 (of these, 5 pursuing a PhD/MD | 28% |
| Phd/MD | 15 | 47% |
| **Nationality** |  |  |
| United States of America | 13 | 41% |
| Nigerian | 5 | 16% |
| Germany | 4 | 13% |
| India | 2 | 6% |
| Albania | 1 | 3% |
| Estonia | 1 | 3% |
| India | 1 | 3% |
| Mexico | 1 | 3% |
| Netherlands | 1 | 3% |
| Pakistan | 1 | 3% |
| Poland | 1 | 3% |
| South Korea | 1 | 3% |
| **Do you have a chronic health condition?** |  |  |
| Yes | 4 | 13% |
| No | 28 | 87% |
| **How many medical visits** |  |  |
| Once a month or more | 2 | 6% |
| Once every three months | 6 | 19% |
| Once a year or less | 24 | 75% |
